# Protection of oral hydrogen water as an antioxidant on pulmonary hypertension

**DOI:** 10.1007/s11033-013-2653-9

**Published:** 2013-08-18

**Authors:** Bin He, Yufeng Zhang, Bo Kang, Jian Xiao, Bing Xie, Zhinong Wang

**Affiliations:** 1 Department of Anesthesiology and SICU, Xinhua Hospital, Shanghai Jiaotong University School of Medicine, Kongjiang Road 1665, Shanghai, 200092 China; 2 Department of Cardiothoracic Surgery, Changzheng Hospital, Second Military Medical University, Fengyang Road 415, Shanghai, 200003 China; 3 Department of Burn, Changhai Hospital, Second Millitary Medical University, Changhai Road 168, Shanghai, 200433 China

**Keywords:** Antioxidant, Hydrogen water, Pulmonary hypertension

## Abstract

This study aimed to explore the protective effect of hydrogen as an antioxidant on monocrotaline (MCT)-induced pulmonary hypertension (PH). Forty-eight SD rats were equally randomized into four groups: SHAM group, MCT group, MCT+Oral-H_2_ group and MCT+Inj-H_2_ group. The results showed that the mean pulmonary arterial pressure, right ventricle weight and right ventricular hypertrophy index in MCT group were significant higher than those in SHAM group; pulmonary inflammatory response, atrial natriuretic factor, 3-nitrityrosine and intercellular adhesion molecule-1 were also increased significantly in MCT group. These indexes were decreased significantly in both MCT+Oral-H_2_ group and MCT+Inj-H_2_ group, which indicate Oral-H_2_ and Inj-H_2_ have similar effects of preventing the development of PH and mitigating RV hypertrophy. The protective effect of hydrogen is associated with its antioxidative ability and action of reducing pulmonary inflammatory response. While Oral-H_2_ is more convenient than Inj-H_2_, Oral-H_2_ may be ideal for clinical use in future.

## Introduction

Pulmonary hypertension (PH) is clinically characterized by progressive increase of pulmonary arterial pressure and right ventricular hypertrophy [1]. It is believed that PH is a severe condition attributable to co-action of multiple etiologies and factors, including genetic susceptibility, immunologic disturbances and environmental stimuli [2–6]. Previous studies mainly focused on the use of vasodilators [7–9], including nitric oxide inhalation, and use of cyclic guanosine monophosphate generating agents, endothelin receptor antagonists and prostatin. However, these vasodilators were unable to radically reverse the progressive increase of PH. It is therefore necessary to find a new strategy that could cure PH effectively and prevent pulmonary vascular resistance from rising progressively [10].

Studies [11–14] have demonstrated that reactive oxygen species (ROS) plays an important role in the occurrence of PH, and those antioxidants are effective in the treatment of PH [15, 16]. However, the use of large doses of non-selective antioxidants may induce hemorrhage and other complications [17, 18], and therefore rational use of selective antioxidants is more advantageous in the treatment of PH. Recent studies [19–21] have shown that molecular hydrogen is a selective antioxidant, and plays a role in protecting against ischemia/reperfusion (I/R) injury through selectively clearing hydroxyl radicals (^·^OH) and peroxynitrite (ONOO-); and hydrogen does not react with other ROS (e.g., H_2_O_2_ and O_2_−), which possess physiological roles. Our previous study [22] also demonstrated that intraperitoneal Inj-H_2_ could protect against I/R injury through selective anti-oxidization. So we established a PH rat model by intraperitoneal injection of monocrotaline (MCT) to see whether hydrogen was also effective in PH treatment.

There are various ways [19–22] to administrate molecular hydrogen as a therapeutic antioxidant, inhalation of hydrogen gas, oral administration of hydrogen water, intraperitoneal or intravenous injection of hydrogen-rich saline. As hydrogen gas is explosive, this might be potential dangerous for clinical application. Besides using Inj-H_2_ in our previous study [22], we also made new modification in production and administration of hydrogen by using Oral-H_2_ in the present study. And we will also compare these two different ways to administrate hydrogen in therapeutic effect on MCT-induced PH.

## Materials and methods

### Animals

Adult SD rats weighing 250–280 g (Experimental Animal Center of the Second Military Medical University, Shanghai, China) were housed with free access to food and water under a natural day/night cycle, and acclimated for 7 days before any experimental procedure. All experimental procedures were approved by the Institutional Animal Care and Use Committee of the Second Military Medical University in Shanghai, China.

### Production of Oral-H_2_ and Inj-H_2_

Oral-H_2_ [20]: The magnesium stick used to produce hydrogen in the study was a plastic-shelled product consisting of metallic magnesium (99.9 % pure) and natural stones in a polypropylene container combined with ceramics (Doctor SUISOSUI^®^, Friendear, Tokyo, Japan). It was able to generate hydrogen when placed in drinking water through the following chemical reaction: Mg + 2H_2_O → Mg(OH)_2_ + H_2_. The hydrogen concentration in the water bottle was sequentially monitored using a hydrogen needle sensor (DHS-001, ABLE, Tokyo, Japan) and maintained at a level between 0.55 and 0.65 mmol and pH 7.7–8.3.

Inj-H_2_ [22]: Hydrogen was dissolved in 0.9 % saline for 6 h under a high pressure (0.4 MPa) to a supersaturated level. Hydrogen saline was stored under an atmospheric pressure at 4 °C in an aluminum bag with no dead volume, sterilized by gamma radiation, and freshly prepared once a week to ensure that the concentration was maintained at 0.6 mmol/L. Gas chromatography was performed to confirm the content of hydrogen in saline.

### Establishment of the in vivo rat model and experimental protocols

Forty-eight SD rats were equally randomized to four groups: SHAM group as the control, MCT group, MCT+Oral-H_2_ group and MCT+Inj-H_2_ group. Animals in SHAM group were administered with Oral-H_2_ only, and the other groups were administered with *intraperitoneal* injection of MCT (80 mg/kg).

Fourteen days later, the mean pulmonary arterial pressure (mPAP) was measured in all the groups. Animals in MCT group were administered with ordinary drinking water thereafter. Those in MCT+Oral-H_2_ group or MCT+Inj-H_2_ group were administered with Oral-H_2_ or Inj-H_2_ respectively.

Additional 14 days later, mPAP was measured again. All animals were then sacrificed, and the heart and lung tissue were removed for morphologic observation and related tests.

### Determination of mPAP

The rats were anesthetized by *intraperitoneal* injection of 300 mg/kg chloral hydrate and fixed in a supine position. The right jugular vein was isolated, through which a PE50 catheter was inserted to the right ventricle (RV) to the right atrium, and finally placed in the pulmonary trunk. The end of the catheter was connected to a pressure sensor to monitor, analyze and record mPAP in a real-time manner.

### Measurement of RVHI

The RV, left ventricle (LV) and septum (S) were weighed separately. The right ventricular hypertrophy index (RVHI) was measured by RV/(LV+S) weight.

### Histopathology of lung injury and RV hypertrophy

*Lung injury* [23] The pulmonary tissues were stained with HE. Morphological lung injury in different groups was assessed by at least two pathologists independently according to the procedures described previously.

*Determination of ANF* Besides RVHI, ANF is another marker for RV hypertrophy. Blood for plasma atrial natriuretic factor (ANF) measurements were collected in heparinized syringes. Plasma was separated by centrifugation and frozen at −70 °C. Plasma ANF was measured by ELISA method (Abcam Inc.) [24].

### Determination of 3-nt and ICAM-1 levels

*Immunohistochemistry* The rats were sacrificed, and the lung tissue was sliced, fixed, paraffin-embedded and dewaxed routinely as described [22]. The primary antibodies for the determination of 3-nitrityrosine (3-nt) and intercellular adhesion molecule-1 (ICAM-1) were products of Abcam Inc., and the secondary antibodies were products of R&D Systems. The prepared immunohistochemical sections were observed in randomly selected four fields (×20), and the number of 3-nt and ICAM-1 positive cells was counted quantitatively.

*Western blot* The protein expression of 3-nt and ICAM-1 was detected similar to our previous study [25]. All the same membranes were probed with β-actin as loading controls.

### Statistical analysis

Quantitative data were expressed as mean ± SD. Differences within the three groups were determined with one-way ANOVA and Student–Newman–Keuls test. *P* < 0.05 was considered statistically significant.

## Results

### MPAP and RVHI measurement

Fourteen days after *intraperitoneal* injection of MCT, mPAP in MCT, MCT+Oral-H_2_ and MCT+Inj-H_2_ groups was 29.6 ± 7.2 mmHg, 28.4 ± 8.5 mmHg and 29.1 ± 8.7 mmHg respectively. They were all significantly higher than that in SHAM group which was 18.6 ± 4.3 mmHg (*P* < 0.05), indicating successful establishment of PH animal model. There was no significant difference in mPAP within MCT group, MCT+Oral-H_2_ group and MCT+Inj-H_2_ group (*P* > 0.05).

After additional 14 days, mPAP and RVHI were measured again. As shown in Table 1, mPAP, RV and RVHI in MCT group were all significantly higher than those in SHAM group (*P* < 0.05). While these indexes were decreased significantly in MCT+Oral-H_2_ group and MCT+Inj-H_2_ group (*P* < 0.05), indicating that both Oral-H_2_ and Inj-H_2_ can alleviate MCT-induced PH. There was no significant difference between MCT+Oral-H_2_ group and MCT+Inj-H_2_ group (*P* > 0.05), which indicate Oral-H_2_ and Inj-H_2_ have similar protective effect on PH.

**Table 1:** General results obtained at day 28

	SHAM group	MCT group	MCT+Oral-H_2_ group	MCT+Inj-H_2_ group
Body weight (g)	321 ± 8.6	275 ± 11.6*	281 ± 13.8*	288 ± 13.4*
Heart weight (g)	0.93 ± 0.042	1.17 ± 0.073*	1.11 ± 0.061*	1.13 ± 0.065*
RV weight (g)	0.18 ± 0.004	0.438 ± 0.051*	0.346 ± 0.041*^※^	0.349 ± 0.040*^※^
LV + S weight (g)	0.751 ± 0.031	0.734 ± 0.021	0.755 ± 0.032	0.745 ± 0.028
RVHI	0.24 ± 0.014	0.59 ± 0.065*	0.45 ± 0.048*^※^	0.47 ± 0.052*^※^
mPAP (mmHg)	19.8 ± 5.1	44.6 ± 7.2*	31.4 ± 6.5*^※^	32.2 ± 6.7*^※^
Plasma ANF (pg/ml)	185.6 ± 53.8	693.7 ± 165.9*	407.2 ± 102.3*^※^	423.4 ± 114.0*^※^

* *P* < 0.05 compared with SHAM group; ^※^ *P* < 0.05 compared with MCT group

### Assessment of RV hypertrophy

PH can induce RV hypertrophy. Besides RVHI, ANF is another marker for RV hypertrophy. Plasma ANF was detected by ELISA method. The results showed that ANF in MCT group was significantly higher than that in SHAM group (*P* < 0.05). While ANF was decreased significantly in MCT+Oral-H_2_ group and MCT+Inj-H_2_ group than in MCT group (*P* < 0.05) (Table 1).

### Determination of 3-nt

**Fig. 1:**
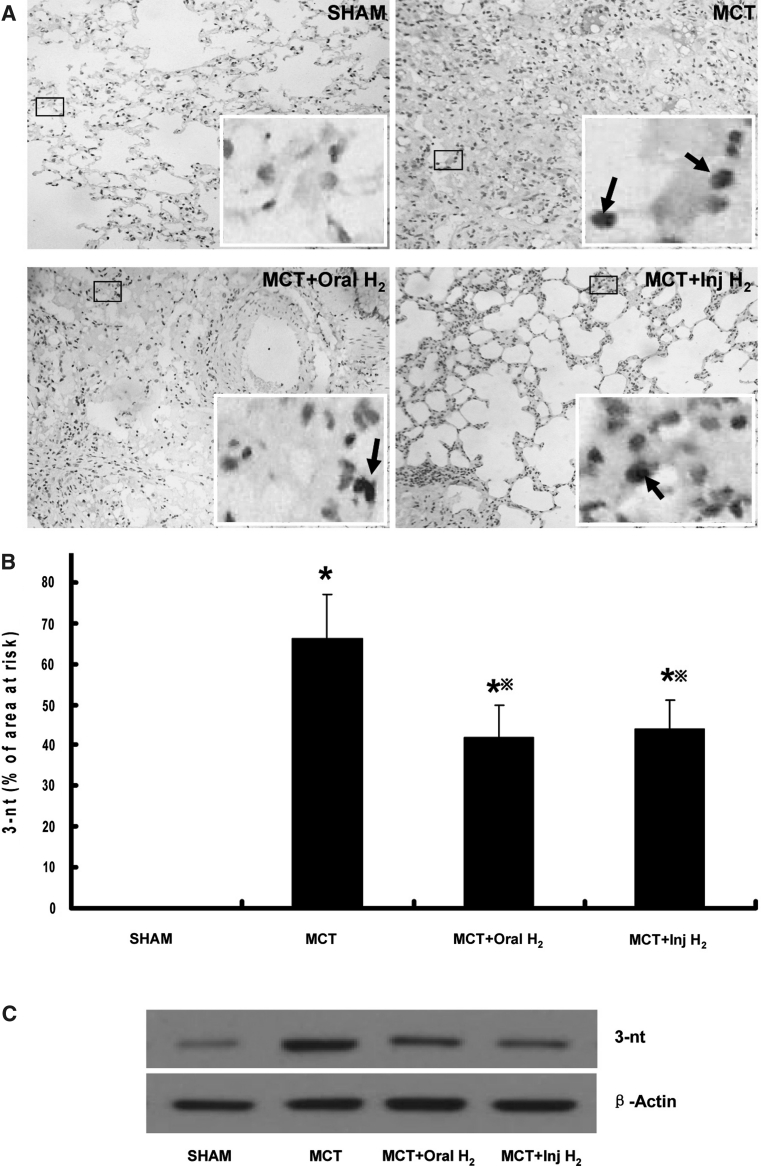
3-nt levels in different groups. **a** Immunohistochemistry. The inset in the lower right corner of the figure showed a section at larger scales, and arrows to show the positive cells. **b** Positive rate. The number of 3-nt positive cells in MCT group was significantly greater than that in SHAM group, and the number of 3-nt positive cells in MCT+Oral-H_2_ and MCT+Inj-H_2_ groups was significantly lower than that in MCT group. **c** Western blot. The protein expression of 3-nt in MCT group was up-regulated than that in SHAM group, while both Oral-H_2_ and Inj-H_2_ can down-regulate the expression of 3-nt. (*P < 0.05 compared with SHAM group; ^※^ *P* < 0.05 compared with MCT group)

Immunohistochemistry for 3-nt was performed on biopsies obtained from the lungs. The number of 3-nt positive cells in MCT group were significantly greater than that in SHAM group, with a positive rate of 66 ± 11 % (*P* < 0.05), indicating that nitrative stress in MCT group was increased. While the number of 3-nt positive cells in MCT+Oral-H_2_ group and MCT+Inj-H_2_ group were significantly lower than that in MCT group, with a positive rate of 42 ± 8 % and 44 ± 7 % respectively (*P* < 0.05), indicating that both Oral-H_2_ and Inj-H_2_ can mitigate the nitrative stress in the course of PH formation (Fig. 1a, b).

The protein expression of 3-nt in different groups was determined by western blot. The protein expression of 3-nt in MCT group was up-regulated than that in SHAM group, while both Oral-H_2_ and Inj-H_2_ can down-regulate the expression of 3-nt (Fig. 1c).

### Determination of ICAM-1

**Fig. 2:**
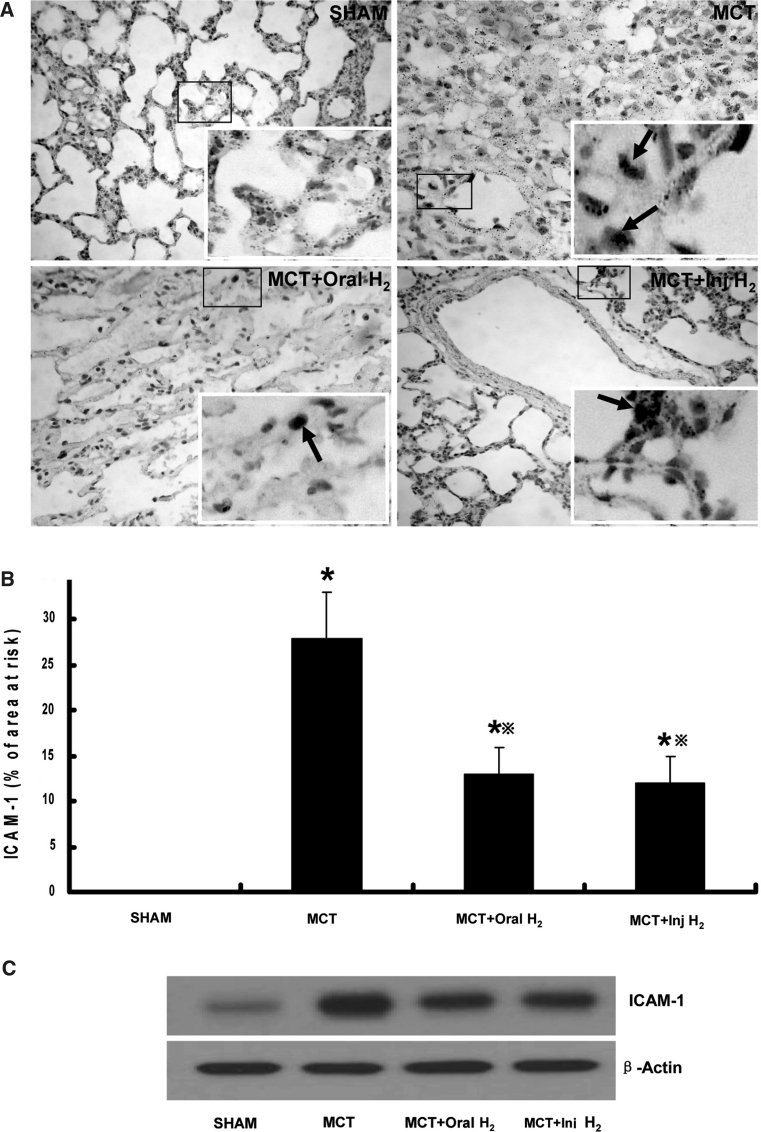
ICAM-1 levels in different groups. **a** Immunohistochemistry. The *inset* in the lower right corner of the figure showed a section at larger scales, and *arrows* to show the positive cells. **b** Positive rate. The number of ICAM-1 positive cells in MCT group was significantly greater than that in SHAM group, and the number of ICAM-1 positive cells in MCT+Oral-H_2_ and MCT+Inj-H_2_ groups was significantly smaller than that in MCT group. **c** Western blot. The protein expression of ICAM-1 in MCT group was up-regulated than that in SHAM group, while both Oral-H_2_ and Inj-H_2_ can down-regulate the expression of ICAM-1. (*P < 0.05 compared with SHAM group; ^※^ *P* < 0.05 compared with MCT group)

Immunohistochemistry for ICAM-1 was performed. The number of ICAM-1 positive cells in MCT group were significantly greater than that in SHAM group, with a positive rate of 28 ± 5 % (*P* < 0.05), implying that ICAM-1 infiltration was increased in MCT group. The number of ICAM-1 positive cells in MCT+Oral-H_2_ group and MCT+Inj-H_2_ group were significantly lower than that in MCT group, with a positive rate of 13 ± 3 % and 12 ± 3 % respectively (*P* < 0.05), suggesting that both Oral-H_2_ and Inj-H_2_ can mitigate ICAM-1 infiltration in the course of PH formation (Fig. 2a, b).

The protein expression of ICAM-1 in different groups was determined by Western blot. The protein expression of ICAM-1 in MCT group was up-regulated than that in SHAM group, while both Oral-H_2_ and Inj-H_2_ can down-regulate the expression of ICAM-1 (Fig. 2c).

### Analysis and scoring of lung tissue injury

**Fig. 3:**
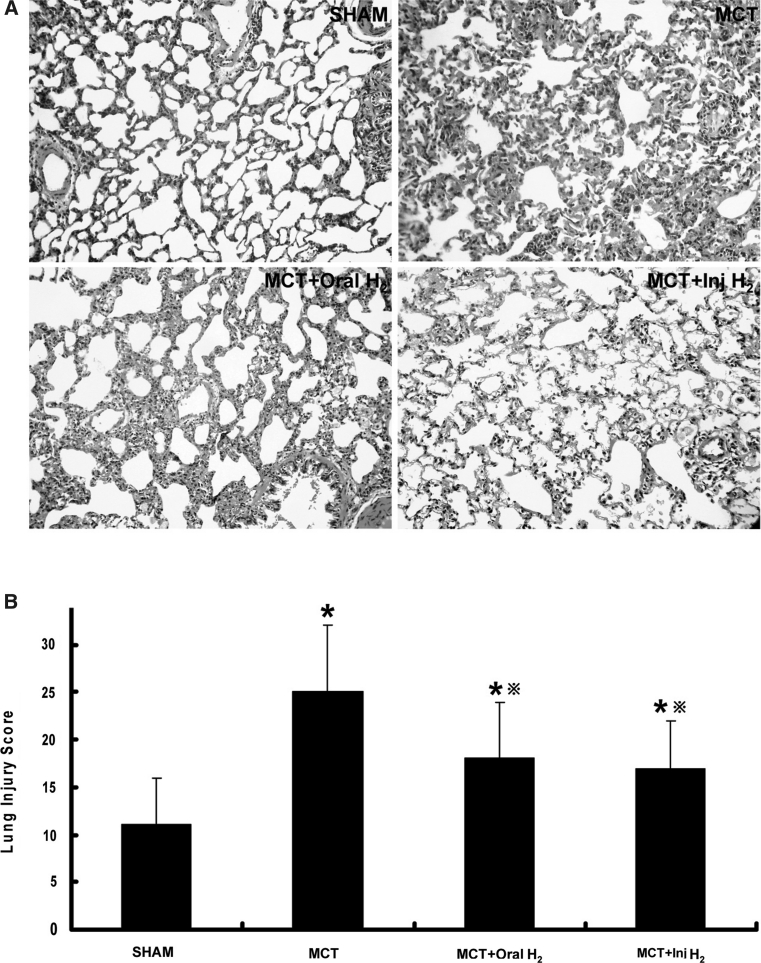
Results of lung tissue injury. **a** HE Staining. In SHAM group, the lung tissue was normal. In MCT group, inflammatory injury to the lung tissue was severe, presenting as fracture of the alveolar septa and infiltration of macrophages and chronic inflammatory cells as indicated by the presence of scattered red blood cells. Media thickening, luminal narrowing, fibroblast proliferation and adventitial inflammatory response in pulmonary arterioles. In the lung tissue sections of MCT+Oral-H_2_ and MCT+Inj-H_2_ groups, the number of inflammatory cells was reduced significantly, and alveolar septa were also reduced significantly as compared with SHAM group. **b** Lung injury score. Semi-quantitative scoring of the lung tissue in different groups showed that the lung tissue injury score of MCT group was significantly higher than that of SHAM group, and the lung tissue injury score of MCT+Oral-H_2_ and MCT+Inj-H_2_ groups was significantly lower than that of MCT group (*P < 0.05 compared with SHAM group; ^※^ *P* < 0.05 compared with MCT group)

There was no significantly abnormal change in lung tissue was observed in SHAM group, while there was significant evidence of inflammatory response in the lung tissue of the rats in MCT group, presenting as fracture of parenchymal cells, infiltration of macrophages and chronic inflammatory cells, and significant thickening of alveolar septa and cavities. Compared with MCT group, these inflammatory responses were significantly ameliorated in both MCT+Oral-H_2_ group and MCT+Inj-H_2_ group (Fig. 3a). The tissue injury score was 11 ± 5 in SHAM group, 25 ± 7 in MCT group, 18 ± 6 in MCT+Oral-H_2_ group and 17 ± 5 in MCT+Inj-H_2_ group, which indicated that tissue injury in MCT group was more severe than that in SHAM group (*P* < 0.05), while that in MCT+Oral-H_2_ group and MCT+Inj-H_2_ group was ameliorated significantly (*P* < 0.05) (Fig. 3b).

## Discussion

Inflammatory response in lung tissue, progressive increase of pulmonary arterial pressure and pulmonary vascular resistance, resultant RV hypertrophy and remodeling are typical pathological presentations of PH [26]. RVHI and plasma ANF were detected, both of which are markers for RV hypertrophy. Both RVHI and ANF were decreased significantly by hydrogen. These results of the present study showed that hydrogen can relieve MCT-induced PH markedly and reverse RV hypertrophy. It was found in our experiment that hydrogen was able to reduce the thickness of alveolar septa and the number of chronic inflammatory cells in the course of PH formation, thus mitigating RV hypertrophy. Knowing that ROS is closely correlated with RV hypertrophy as demonstrated in previous studies [27], and that hydrogen can clear ROS, we suppose that the mechanism of hydrogen in mitigating RV hypertrophy is via selectively clearing ROS and reducing pulmonary arterial pressure. As a result, not only RV load was reduced but RV hypertrophy was mitigated.

Previous studies have demonstrated that chronic inflammatory response plays an crucial role in the development and progression of PH [2], and that increased ROS production is inextricably associated with chronic inflammatory response [28], which is also confirmed by the fact that antioxidants are effective in the treatment of PH [15, 23]. Our previous experiment [22] demonstrated that molecular hydrogen had an anti-inflammatory effect on I/R injury. The present study further confirmed that molecular hydrogen was able to mitigate the infiltration of inflammatory cells in the course of MCT-induced PH formation, thus relieving inflammatory response. ICAM-1 is one of the main adhesion molecules mediating neutrophil adhesion, and is regulated by such cytokines as TNF-α and IL-1b. After cytokine regulation, ICAM-1 is up-regulated rapidly, thus progressively aggravating the inflammatory response. If ICAM-1 could be reduced effectively, inflammatory response in the course of pathological injury would be mitigated [29, 30]. It was found in the present study that hydrogen could reduce ICAM-1 level in the course of PH formation and infiltration of chronic inflammatory cells in local lung tissue, thus relieving the severity of PH.

It was also found in our study that improvement of PH was significantly correlated with 3-nt level. 3-nt is a specific metabolic product of ONOO-, reflecting the level of nitrative stress in vivo [31]. The fact that hydrogen could effectively relieve MCT-induced PH implies that ONOO- plays an important role in the development and progression of PH. There are various types of ROS produced in vivo, including hydroxyl free radical, ONOO-, peroxide and superoxide anion. But which ROS actually plays a role in the development and progression of PH remains unclear. Previous related studies mainly focused on the use of antioxidants, and few studies have focused on which ROS or how many ROS playing the role. The present study demonstrated that hydrogen could reduce 3-nt level markedly and mitigate the pathological process of PH, which indirectly proves that ONOO- may participate in PH formation. It is noteworthy that some studies [32] also found that ONOO- played an important role in chronic inflammation, which may explain why reducing ONOO- level could mitigate the severity of PH.

Although studies showed that some antioxidants could effectively reduce PH, few of them have been used clinically. Imbalance in the endogenous redox state due to nonselective clearance is supposed to be the main reason for failure of some antioxidants in the treatment of PH [33, 34]. Selective clearance is therefore the rational method for effective treatment of PH. It has been proved [19] that molecular hydrogen is a selective antioxidant, which can selectively clear ^·^OH and ONOO-. And hydrogen does not react with other ROS, which possess physiological roles. In addition, due to the small molecular weight, hydrogen can easily enter cell membrane and organelle membrane. These are the advantages of hydrogen in playing antioxidative effect on PH.

In our previous study [22], we used *intraperitoneal* Inj-H_2_ as an antioxidant. In the present study, we made new modification in production and administration of hydrogen by using oral hydrogen water (Oral-H_2_) generated by magnesium stick. The results showed Oral-H_2_ and Inj-H_2_ have similar effect of preventing the development of PH and mitigating RV hypertrophy.

As shown in Table 2, we also compared between the two different ways to administrate hydrogen (Oral-H_2_ and Inj-H_2_). Unlike Inj-H_2_, The administration and absorption of Oral-H_2_ are non-invasive and physiological. As hydrogen can also be produced in the human intestinal tract, it is safe to drink a certain amount of Oral-H_2_. Inj-H_2_ needs high pressure (0.4 MPa) to produce, and is stored under atmospheric pressure at 4 °C in an aluminum bag without dead volume. While the production and storage of Oral-H_2_ are more convenient, just under normal pressure and normal temperature. The release time of Inj-H_2_ is transient, need freshly preparation every week to ensure a constant concentration of greater than 0.6 mM. While Oral-H_2_ can release hydrogen continuously as long as magnesium stick not depleted. And the cost of Oral-H_2_ is only 10–15 % price of Inj-H_2_. Oral-H_2_ is more convenient, less costly and longer release time than Inj-H_2_, and drinking water containing a high concentration of hydrogen is a novel, safe and potent approach for the prevention and treatment of PH, so Oral-H_2_ may be more ideal for patients in future, as it can be administered without changing their lifestyle.

**Table 2:** Comparison between two different ways to administrate hydrogen

	Oral-H_2_	Inj-H_2_
Administration & absorption	Oral administration is non-invasive; gastrointestinal tract absorption is physiological	Injection is invasive; intraperitoneal or intravenous absorption is non-physiological
Production & storage	Convenient; normal pressure; normal temperature	Need more higher pressure to produce; storage at 4 °C; need special instrument to store: aluminum bag without dead volume
Release time	Continuous; release hydrogen as long as magnesium stick not depleted	Transient; need freshly preparation every week to ensure a constant concentration of greater than 0.6 mM
Cost	Less; only 10–15 % price of Inj-H_2_	More
Clinical application	Ideal and convenient for patients without changing their lifestyle	Need injection everyday

In summary, the results of the present experiment showed that hydrogen can reduce PH, mitigate RV hypertrophy by reduce RVHI and ANF in a MCT-induced PH rat model. In addition, hydrogen could reduce inflammatory response and 3-nt and ICAM-1 levels in the course of PH formation, suggesting that the therapeutic effect of hydrogen in the treatment of PH is closely correlated with antioxidative stress. While Oral-H_2_ is more convenient than Inj-H_2_, so Oral-H_2_ may be more ideal for clinical use in future. Next step, we plan to make in-depth study on the mechanism of hydrogen in reducing PH, especially the influence of hydrogen on cytokines that are closely associated with PH.
